# YOLOv5-Atn: An Algorithm for Residual Film Detection in Farmland Combined with an Attention Mechanism

**DOI:** 10.3390/s23167035

**Published:** 2023-08-08

**Authors:** Ying Lin, Jianjie Zhang, Zhangzhen Jiang, Yiyu Tang

**Affiliations:** 1 College of Software, Xinjiang University, Urumqi 830091, China; 107552104356@stu.xju.edu.cn; 2 College of Mechanical Engineering, Xinjiang University, Urumqi 830017, China

**Keywords:** object detection, YOLOv5, residual film, attention mechanism

## Abstract

The application of mulching film has significantly contributed to improving agricultural output and benefits, but residual film has caused severe impacts on agricultural production and the environment. In order to realize the accurate recycling of agricultural residual film, the detection of residual film is the first problem to be solved. The difference in color and texture between residual film and bare soil is not obvious, and residual film is of various sizes and morphologies. To solve these problems, the paper proposes a method for detecting residual film in agricultural fields that uses the attention mechanism. First, a two-stage pre-training approach with strengthened memory is proposed to enable the model to better understand the residual film features with limited data. Second, a multi-scale feature fusion module with adaptive weights is proposed to enhance the recognition of small targets of residual film by using attention. Finally, an inter-feature cross-attention mechanism that can realize full interaction between shallow and deep feature information to reduce the useless noise extracted from residual film images is designed. The experimental results on a self-made residual film dataset show that the improved model improves precision, recall, and mAP by 5.39%, 2.02%, and 3.95%, respectively, compared with the original model, and it also outperforms other recent detection models. The method provides strong technical support for accurately identifying farmland residual film and has the potential to be applied to mechanical equipment for the recycling of residual film.

## 1. Introduction

Mulching film is a plastic film covering the soil surface. As a mature agricultural cultivation technology, mulching film technology has the functions of maintaining soil moisture, improving light conditions, increasing soil fertility, inhibiting weed growth, preventing pests and diseases, and so on [1,2]. It has played a positive role in improving the farmland planting environment and increasing crop yields, and has become one of the most important planting methods in China’s agricultural production. With the gradual increase in the use of mulching film and the continuous expansion of the coverage area [3], the shortcomings of mulching film are becoming increasingly exposed. Mulching film is easy to break, difficult to degrade, and difficult to recycle, resulting in an increasing amount of film residue on farmland. Residual film disrupts soil continuity; reduces soil water content, air permeability, and fertility levels; and causes soil consolidation, thus affecting normal crop growth and development [4,5]. Currently, discarded mulching film recycling can be divided into manual picking and mechanical recycling. Manual recycling is labor-intensive, time-consuming, and inefficient, and farmers are not highly motivated to complete the task of recycling large areas of residual film. Mechanical recycling can effectively overcome the disadvantages of manual recycling, improve labor productivity and the residual film collection rate, and meet the requirements for discarded mulching film recycling [6]. In recent years, researchers have developed many forms of residual film recycling machines [7,8,9,10], but residual film recycling machines are almost useless for small sizes of film. In view of the shortcomings of the existing residual film recycling machines, this paper proposes the use of target detection technology to identify and locate residual film to provide a theoretical basis for the realization of the development of an intelligent residual film picking machine. The study of algorithms for accurately detecting farmland residual film is the key to developing intelligent residual film recycling machines and improving the residual film recovery rate.

In recent years, image processing techniques have been widely used in agriculture [11,12], and researchers have used these techniques to identify and detect mulching film. Early image processing methods were based on traditional machine learning, including using some image processing operators to extract the features of an image and using a support vector machine (SVM) to perform classification to obtain the targets in the image. In the early stage, when image processing technology was not mature enough, Jiang et al. [13] used the histogram threshold segmentation method to determine the threshold, combined with edge detection and region filling to separate the residual film image. Zhu et al. [14] used unmanned aerial vehicle (UVA) remote sensing as a platform to obtain images of mulched farmland in Yunnan Province. They extracted the area of mulched farmland by combining the morphological algorithm and area threshold segmentation algorithm. Fu et al. [15] proposed a real-time extraction method to obtain film-covered farmland from complex mixed ground surfaces based on multi-source remote sensing data. Hasituya et al. [16] combined the spectral and texture features based on Landsat-8 remote sensing data to classify and identify mulching film in farmland using SVM, a maximum likelihood classifier, and a minimum distance classifier, respectively. Lu et al. [17] used spectral, backscatter, index, and texture features from Sentinel-1 and Sentinel-2 to classify mulching land cover and other land cover types.

With the continuous update of computer technology, deep learning has been gradually applied as a new research direction in machine learning, contributing to artificial intelligence’s realization. Deep learning technology has become an indispensable component for the development of modern agriculture, providing an essential technical force for the realization of agricultural informatization and automation and helping to improve agricultural intelligence. Sun et al. [18] used UAVs to collect images of greenhouses and film-covered farmland in Wangfu Township, Chifeng City. They constructed a full convolutional network (FCN) combined with the multi-scale feature fusion method to achieve the rapid recognition of film-covered farmland images. Yang et al. [19] showed that the deep semantic segmentation model is more effective in recognizing film-covered farmland by comparing two deep semantic segmentation methods, SegNet and FCN, and the traditional classification method, SVM. Ning et al. [20] proposed an improved semantic segmentation model, DeepLabv3+, applicable to determine whether farmland is mulched or not to achieve effective segmentation of film-covered farmland in UAV multispectral remote sensing images. Zhang et al. [21] improved the Faster R-CNN convolutional neural network and applied it to the recognition and detection of residual film in farmland. The improved model uses a dual-threshold algorithm instead of the traditional single-threshold algorithm and selects ResNet50 with a residual network structure as the backbone feature extraction network.

In summary, most of the existing farmland film recognition and detection methods are based on remote sensing images from the use of farmland film mulching as the research object. At this time, the film has the characteristics of a large coverage area and distinctive features [22]. Spectral imaging technology is used to identify and detect farmland film by combining spectral characteristics and image processing technology. However, there are few studies on residual film in the late stage of farmland film mulching, i.e., after the harvest of crops. The difference in texture and shape between residual film and bare soil is not obvious due to wind and rain erosion, and residual film is of various sizes and shapes [23]. To address the above problems, this paper proposes a farmland residual film detection algorithm combined with the attention mechanism. The main contributions are as follows:

1. In order to verify the improved model, a small and medium sized dataset for the detection of residual film is made by using residual film images captured in the natural environment;
2. A two-stage training method for the residual film task was designed to improve the problem of less residual film data, effectively improving the model’s accuracy and generalization;
3. An adaptive multi-scale feature fusion module (ASFF) is proposed, which adds learnable adaptive weights to allow the model to adaptively select the appropriate size of feature maps, effectively solving the problem of model missed detection due to the different sizes of residual film;
4. An inter-feature cross-attention (FCA) module is proposed to improve the existing skip-connection mechanism of the model. The correlation feature information of the shallow layer and deep layer is effectively used, and the interference noise in the shallow layer is eliminated.

## 2. Dataset

### 2.1. Data Acquisition

**Figure 1:**
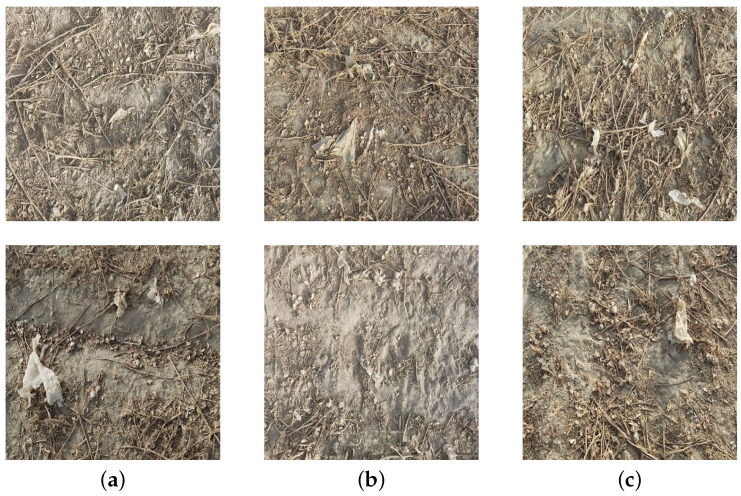
Example of the dataset. (**a**) Different light intensity. (**b**) Different soil moisture. (**c**) Different amounts of residual film.

The farmland residual film images were collected in the cotton planting area of Yuli County, Bayingoling Mongolian Autonomous Prefecture, Xinjiang Uygur Autonomous Region. The residual film on the surface of the farmland after the operation of a 11JCM-300 residual film recycling machine was used as the research object. A Huawei Mate 40 Pro cell phone was used to acquire the residual film images with a resolution of 4092 × 3072 pixels. During the acquisition, the camera lens was parallel to the ground at a height of about 80 to 100 cm from the ground. Non-conforming images such as blur and jitter were removed, and a total of 2000 residual film images were acquired. The sample contained residual film images with different shooting angles, lighting conditions, and soil moisture content, thus ensuring the diversity of images. The residual film images taken under different environments are shown in Figure 1.

### 2.2. Data Annotation

**Figure 2:**
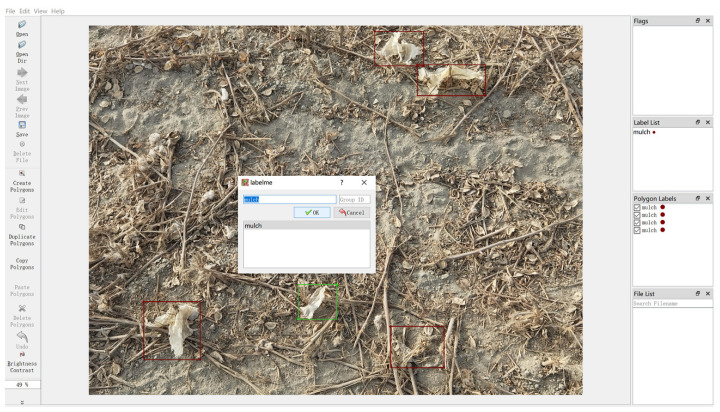
Labeling of residual film.

The training of the residual film image dataset is inseparable from the location data and the category data of the residual film in the image, which requires labeling of the residual film image. The labeling software Labelme (version 4.5.6) was used to label the residual film images one by one, and each labeled residual film target was given a mulch label, as shown in Figure 2. The software stored the labeled residual film border location data in a separate json file. After all the residual film images were labeled, the dataset was divided according to a ratio of 4:1, with 1600 training set images and 400 validation set images. Then, we wrote a script to integrate the label information of all data sample json files into the respective json files of the training set and validation set, according to the format of the COCO dataset [24].

### 2.3. Data Augmentation

**Figure 3:**
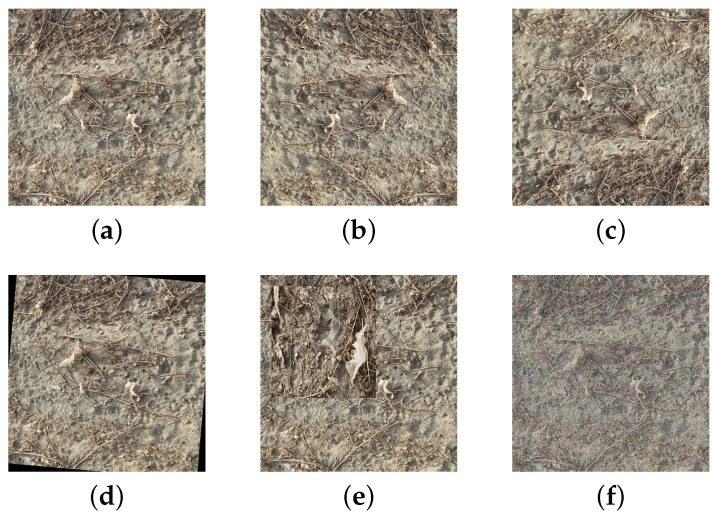
Examples of data augmentation. (**a**) Original image. (**b**) Horizontal flip. (**c**) Diagonal flip. (**d**) Random rotation. (**e**) Image mosaic. (**f**) Gaussian noise.

The dataset of the residual film images is enriched and balanced to better extract the features of the residual film, improve the model’s generalization ability, and reduce the workload of manual annotation. In the training process, online data augmentation was performed on the labeled residual film image dataset, and the dataset was expanded by randomly rotating the image by 5 to 10 degrees, randomly cropping the image, image stitching, image flipping, Gaussian noise, and other data processing methods. Examples of data augmentation are shown in Figure 3.

## 3. Method

### 3.1. YOLOv5

**Figure 4:**
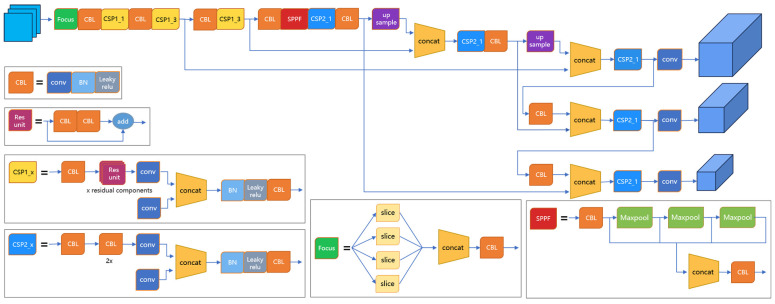
Structure of the YOLOv5 model.

YOLOv5 is a classical single-stage target detection model that requires only one forward propagation to complete the target detection task. It is simpler and more effective than traditional two-stage target detection models such as Faster R-CNN [25] and SSD [26]. The YOLOv5 model consists of Input, Backbone, Neck, and Head. The structure is shown in Figure 4. The input is mainly responsible for preprocessing the input image, and proposes an adaptive anchor calculation and adaptive image scaling method. Backbone is used to extract some generic feature representations, and YOLOv5 uses the CSPDarknet53 structure as the backbone network. The Neck is responsible for the processes of feature fusion and feature processing, using feature pyramid network (FPN) [27] and path aggregation network (PAN) [28] structures to enhance the diversity and robustness of features further. The Head is mainly responsible for predicting the feature map, outputting the target object’s location and category information, and filtering and processing the prediction results.

### 3.2. An Improvement of the YOLOv5 Model: YOLOv5-Atn

**Figure 5:**
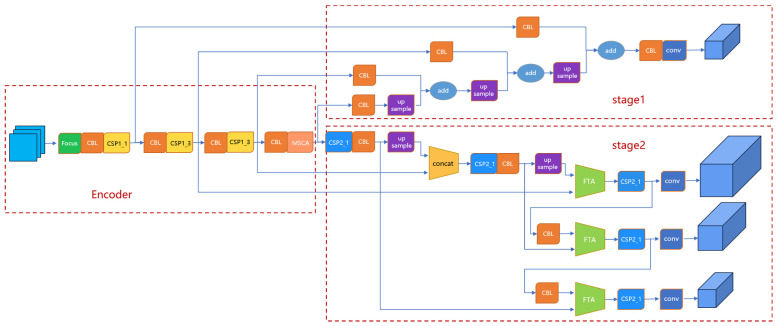
Structure of the YOLOv5-Atn model.

An improved scheme was proposed to address the problem of detecting residual film in deep learning. Based on the YOLOv5 classical network, the original spatial pyramid pooling (SPPF) was replaced with an adaptive multi-scale fusion module designed for residual film features, and the model’s recognition of small residual film targets was enhanced by using attention. When combining deep and shallow features, an inter-feature cross-attention mechanism applicable to convolutional networks was proposed to extract high-quality feature information and reduce the useless noise extracted from the residual film images. The training process was also optimized, and a two-stage training strategy was proposed to strengthen the model’s ability to understand the residual film objects with fewer samples. The improved network structure is shown in Figure 5, and each improved part will be explained in detail in the following subsections.

#### 3.2.1. Two-Stage Training Strategy

Due to the environmental conditions, labeling cost, and other factors, we obtained relatively fewer labeled residual film samples. The limited amount of residual film data cannot cover different residual film in real-world scenes, and the lack of diverse residual film leads to the limited contextual understanding ability of the model. When the input image is significantly different from the training data, the model does not have good generalization. At the same time, the limited data will lack some representative residual film data, and the model cannot understand residual film well, which leads to missed detection and wrong detection by the model.

Under this condition, in order to make the model converge normally and ensure its generalization, the pre-training weights obtained after a large amount of data training are added to reduce the training difficulty of the model and improve the accuracy. YOLOv5 provides the model weights on the COCO dataset, and using this pre-trained weight to fine-tune the residual film task can help the model converge well. The COCO dataset contains 80 categories in natural scenes, covering almost all common objects in life. However, for this particular residual film target detection task, residual film is often the background class in natural scenes, which is the part ignored by image feature extraction. Therefore, directly using the model weights trained by YOLOv5 on the COCO dataset as the pre-training weights for this task cannot obtain the best results.

Therefore, this paper designed a two-stage training strategy for uncommon classes. In the first stage, an improved masked image modeling (MIM) strategy was used to reconstruct only the residual film region in the image, and the original YOLOv5 pre-training weights were replaced by the encoder weights trained in the first stage for the second stage of training. In this way, it could not only reduce the problem of poor model generalization caused by the small number of samples in the dataset, but also solve the problem of difficult model convergence caused by the lack of appropriate pre-trained model weights.

The first stage of model training was carried out by referring to the self-supervised training approach. Through image reconstruction, some regions on the image were masked off randomly, and the final goal of the training was to recover the masked-off regions. For the selection of the mask area, the Box parameter in the label was used as the region limit, and only the residual film part of the image was masked. The area of residual film was divided into 64 patches of equal size, from which 50% of the patches were randomly selected for masking. By this reconstruction, the encoder could pay more attention to the residual film information in the image. The first stage and the second stage shared the same encoder. The decoder design of the first stage referred to the U-Net [29] structure, selected four simple upsampling layers, and realized the reconstruction of the image mask part using skip connection. The purpose of the first stage was to pre-train an encoder that could fully extract the residual film information for the target detection task of the second stage. The second stage was the target detection of residual film objects, and the decoder part of the second stage was consistent with the YOLOv5 decoder structure.

#### 3.2.2. ASFF

The feature information in the image can be extracted well using the image encoder. The encoder’s shallow features refer to the input data’s low-level features, such as edges and textures. In contrast, the deep features are the high-level features obtained by multi-layer neural network learning, which can better represent the semantic information of the data. In the structure of YOLOv5, the high-level semantic features obtained from the encoder go through SPPF to obtain multi-scale feature maps. The core structure of the SPPF layer downsampling is MaxPooling, which can quickly achieve multi-scale feature extraction, but only extracts the maximum values, lacking the most core learnability. For the residual film detection task, the simple pooling operation cannot effectively capture the interrelationship between residual film with multi-scale features. Therefore, atrous convolution was used to replace pooling to achieve the same multi-scale feature extraction function by setting different intervals.

Smaller residual film objects require smaller receptive fields, and larger residual film objects require larger receptive fields. The consistent single receptive field is difficult to effectively identify objects with significant differences in residual film size. The use of fixed dilated rates in extracting multi-scale features ignores the scale of residual film itself. It also leads to a mismatch between the receptive field of the extracted feature maps and the scale of the residual film object. Therefore, a self-attention scale selection module is designed based on the difference of receptive fields with different scale feature maps. The model can choose the appropriate scale to complete subsequent recognition tasks through self-learning. According to the scale of the residual film object, the feature maps with matching receptive fields will be enhanced, while other feature maps will be suppressed [30].

**Figure 6:**
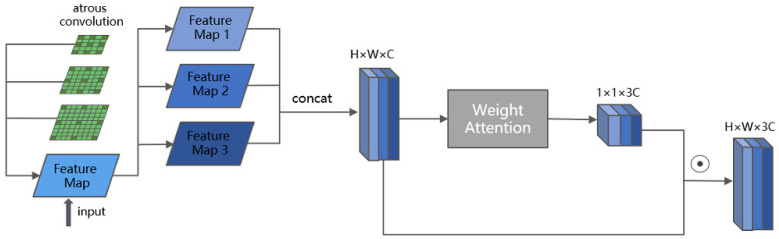
Structure of ASFF.

The specific approach was first to obtain a series of multi-scale features through atrous convolution, and then through the self-attentive selection module. A set of channel dimension weights corresponding to the scales were obtained, and the weights would be larger for the scales suitable for the residual film target. The weights were then combined with the multi-scale features, as shown in Equation (1). The process of self-attention calculation is shown in Equation (2). Since the weight score is only related to itself and is not disturbed by global information, the feature evaluator chooses 1 × 1 convolution. Firstly, the feature was rated by the feature evaluator. Then, the weight scores were normalized by the BatchNormal layer, Relu layer, and Sigmoid layer, and global feature pooling was used to integrate the pixel-level feature weights of each scale. In this way, the model could pay more attention to the selected appropriate scale features in the decoder part, as shown in Figure 6.

(1) Fw=Fs×WeightAtn

(2) WeightAtn=GAP(Relu(BN(Conv(Fs))))

where Fs denotes multi-scale features, Fw denotes multi-scale features filtered by attention weight score, Conv(·) denotes convolution operation with 1 × 1 convolution kernel size, BN(·) is Batch Normal operation, and GAP(·) is global feature pooling operation.

#### 3.2.3. FCA

YOLOv5 adds a skip connection method in the decoding process, which combines the shallow information extracted by the encoder and the deep information extracted by the decoder to obtain richer semantic information. Observing the residual film image data shows that the residual film objects exhibit strong spatial correlations in space. Although this method of skip connection can make good use of the image feature information extracted by the network, it also introduces a large amount of noise in space for a binary classification task like residual film detection. This is because the shallow features of the encoder extract mainly information such as style and contour. Since residual film is similar in shape to the interference objects on the land surface, it is highly susceptible to noise generation.

Therefore, this paper improved the skip connection method to solve the problem of noise introduction. The general skip-connection method directly performs a concat operation on the shallow and deep feature vectors to compose a new feature vector containing rich speech information. If deep and shallow features are simply combined, invalid shallow features may cause the model to detect wrong. The high-level semantic information of the deep layer is utilized to guide the shallow layer information before combining. The residual film feature information in the shallow layer that matches the deep semantics will be selected, which can effectively reduce the effect of useless noise. Referring to the cross-attention mechanism in the Transformer [31] structure, the simple concat operation was modified to a matrix operation of shallow and deep features. The deep features were used as the Query, and the noise was filtered through the cross-attention module to obtain the effective information that the shallow features can provide to the deep features. Due to the large size of the feature map in the convolutional network, the direct introduction of the fully connected layer and matrix cross product operation into the convolutional network of the decoder would cause problems such as gradient explosion during the backpropagation of the network. Therefore, the convolutional network structure was used to realize the information interaction between the two features while ensuring that the core idea of the cross-attention mechanism remains unchanged.

**Figure 7:**
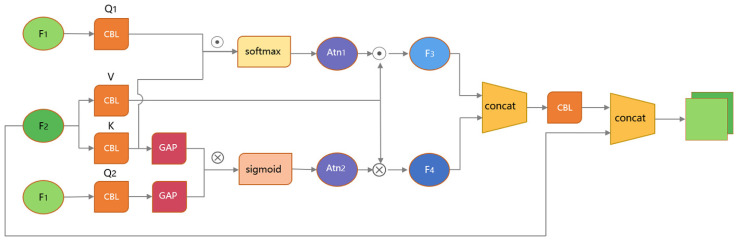
Structure of FCA.

The specific process is shown in Figure 7. Firstly, the deep feature $F_{1}$ of the decoder was mapped by 1 × 1 convolution into spatial query $Q_{1}$ and channel query $Q_{2}$, which were used as conditions for filtering the shallow information. Then, the encoder shallow feature $F_{2}$ was mapped by 1 × 1 convolution into key-value pairs *K* and *V*, which denoted the representation information and specific semantic values of the shallow features, respectively. $Q_{1}$ and *K* were used through the dot multiplication of features to obtain the spatial feature attention weight matrix $Atn_{1}$, as in Equation (3), for spatial-level information filtering. $Q_{2}$ and *K* were used to do the cross product and other operations through the channel feature information after global pooling to obtain the channel feature attention weight $Atn_{2}$, as in Equation (4), for information filtering at the channel level. Then, the attention weight matrix interacted with *V* to extract the effective information in the shallow features. With the help of the attention weight matrix, the shallow features were filtered to remove the noise. Finally, the filtered shallow features were combined with the deep features to realize the interaction of shallow and deep feature information.

(3) $$Atn_{1}=Softmax\left(Mul\left(Q_{1},K\right)\right)$$

(4) $$Atn_{2}=Sigmod\left(MatMul\left(Q_{2},GAP\left(K\right)\right)\right)$$

where $Q_{1}$, $Q_{2}$, *K*, and *V* represent the feature representations obtained by 1 × 1 convolutional mapping of shallow and deep features, Mul(·) represents the dot product, MatMul(·) represents the cross product, and GAP(·) represents the global pooling.

## 4. Experiments

### 4.1. Experimental Setup

The experiments in this paper were performed on a server with the Ubuntu operating system and CUDA Vision 11.2, and the graphics card was an Nvidia GeForce RTX 3090Ti, with 24 GB video memory. The relevant programming languages and deep learning frameworks were Python 3.7, nvcc 11.0, torch 1.8.0, and torchvision 0.9.0.

The experiments were performed on a residual film dataset consisting of photographs of residual film taken in the natural environment and manually labeled. The dataset has a total of 2000 residual film images, divided in a ratio of 4:1 to obtain a training set of 1600 images and a test set of 400 images. The residual film data were enhanced using an online data enhancement strategy.

In the training process, the network was trained in two stages. In the first stage, the target detection decoder was frozen, and the image encoder and image generator were trained by randomly masking 60% of the residual film objects. The epoch size for training was set to 100, the batch size was set to 32, the initial learning rate was 0.0001, and the warmup was set to 1000 iterations. In the second stage, the image generator was frozen, and the image encoder and target detection decoder were trained. The training epoch size was set to 30, the batch size was 16, the initial learning rate was 0.001, and the warmup was set to 1000 iterations. The training optimizer for both stages used the AdamW optimizer, and the learning rate tuning strategy was the cosine annealing strategy.

### 4.2. Evaluation Indicators

In this paper, precision (P), recall (R), mean average precision (mAP), and F1 score were used as evaluation criteria to evaluate the detection performance of the model. The precision indicates the proportion of data detected as positive samples that are true positive samples. The recall indicates the proportion of the total positive samples that the model correctly detects as positive. Higher precision means that a higher percentage of the model’s detection results are residual film targets. The recall indicates the proportion of correctly detected positive samples by the model out of the total positive samples. Higher recall means the model can detect as many residual film targets as possible. The F1 score considers recall and precision equally important, and is the harmonic mean of precision and recall [32]. The precision mean (AP) can be obtained from the geometric area surrounded by the P–R curve formed by precision, and recall and is calculated using integration. The mAP is the average of the AP of all categories and represents a comprehensive measure of the average accuracy of the detected objects. Higher mAP shows that the model has better detection performance on different categories. Like COCO, we chose two IoU thresholds of 0.5 and 0.5∼0.95:0.05 (0.05 means the step size) to measure the model detection performance under different conditions, denoted as mAP and mAP@0.5:0.95 [33]. The formulas are as follows:

(5) $$\mathrm{Precision}=\frac{\mathrm{TP}}{\mathrm{TP}\;+\;\mathrm{FP}}$$

(6) $$\mathrm{Recall}=\frac{\mathrm{TP}}{\mathrm{TP}\;+\;\mathrm{FN}}$$

(7) $$F1=\frac{2\times \mathrm{Precision}\times \mathrm{Recall}}{\mathrm{Precision}\;+\;\mathrm{Recall}}$$

(8) $$\mathrm{AP}=\int_{0}^{1}P(r)\mathrm{dr}$$

(9) $$\mathrm{mAP}=\frac{\sum_{i}^{K}\mathrm{AP}i}{K}$$

where TP is the number of samples whose detection result is positive and are actually positive. FP is the number of samples whose detection result is positive but are actually negative. FN is the number of samples whose detection result is negative but are actually positive. P(r) is the value of P corresponding to R on the P–R curve, and K is the number of all categories.

FLOPs and model size are used to measure the model’s computational complexity. FLOPs refer to the number of floating-point operations in the model [34]. Higher FLOPs mean higher computational complexity of the model, which may require more computational resources. The larger model size usually corresponds to a more complex model that requires more storage space.

FPS and inference time are used to measure the detection speed of the model. FPS refers to the number of images the target network can detect per second. Higher FPS indicates that the model has lower computational complexity and can process data faster. Longer inference time may mean that the model has higher computational complexity and requires a longer inference time to process the input data.

## 5. Results

### 5.1. Ablation Experiments

In order to better verify the effectiveness of the module, experiments were conducted on combining different modules on a residual film dataset. The experimental parameters were kept consistent during the first stage of training. In the second stage, ablation experiments were performed by adding only the ASFF module, only the FCA module, and both, respectively. At the same time, we also verified the comparison between the two-stage training and the single-stage training loaded with the original pre-training weights, and the results of the experiments are shown in Table 1. As can be seen from the table, the two-stage training improved the mAP by 1.08% compared to the original single-stage. This indicated that a better encoder could be obtained through the first stage of training to extract the residual film feature information for the second stage of the target detection task. After adding the ASFF module to the model, the mAP was 1.88% higher than the two-stage training without any module improvement. This indicated that the model could better perceive different sizes of residual film objects through the multi-scale feature fusion module with adaptive weights, effectively solving the missed problem of small targets of residual film. Similarly, mAP improved by 1.94% after adding FCA modules to the model, mainly due to the reduction of noise interference by filtering shallow information through the cross-attention mechanism. When the two modules were combined, the mAP increased by 2.87% to 79.03%, achieving excellent results. The experiments showed that the two modules do not lead to precision degradation due to the restrain but are independent of each other. The feature information of residual film with different receptive fields was effectively extracted, and the shallow and deep features were fully utilized to complete the recognition and decoding of the target object.

### 5.2. Comparison Experiments

In order to demonstrate the excellent detection effect of the YOLOv5-Atn model, comparative experiments are conducted with the classical target detection networks and the latest YOLO models, including Faster R-CNN, YOLOv3, YOLOv5, YOLOv7, and YOLOv8. The classical and improved models were trained on the manually labeled residual film datasets. To ensure the experiment’s credibility, the models were trained and verified under the same configuration environment, each model was trained three times, and the best result was taken as the comparison indicator. A comparison of the detection effect of different models is shown in Table 2, and a comparison of the complexity of different models is shown in Table 3. It could be seen that the mAP of Faster R-CNN, as a classic two-stage target detection network, was significantly lower than that of the mainstream one-stage YOLO series of target detection models. As a classic model of the YOLO series, YOLOv3’s mAP was 13.1% higher than that of the Faster R-CNN model, but the mAP was 2.48% lower than the YOLOv5 model. By adding the ASFF module and FCA module, the improved network improves the feature extraction capability of residual film, and the accuracy rate, recall rate, and mAP are, respectively, increased by 5.39%, 2.02%, and 3.95% compared with the original model. Moreover, compared with YOLOv7 and YOLOv8, the proposed model has improved in precision, F1 score, mAP, detection speed, and other aspects. Overall, the YOLOv5-Atn algorithm not only has good detection accuracy, but also has good detection speed to meet real-time requirements.

**Figure 8:**
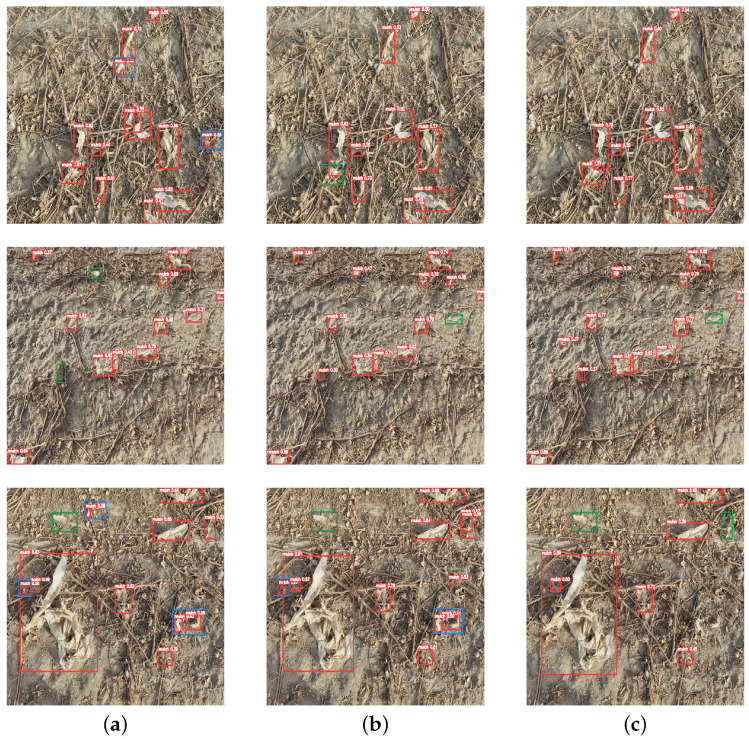
Visualization renderings of residual film images. (**a**) YOLOv5, (**b**) YOLOv8, (**c**) YOLOv5-Atn.

The visualization renderings of several residual film images on the original YOLOv5 model, the improved YOLOV5-Atn model, and the latest YOLOv8 model were selected, as shown in Figure 8. The blue box indicates missed detection, and the green box indicates wrong detection. In the first row, the YOLOv5 model incorrectly detected cotton as residual film, and YOLOv8 has residual film that was not detected. However, YOLOv5-Atn had no missed or wrong detection problems, and the detection effect was significantly higher than the other two models. In the second row, all three models had different degrees of missed detection problems, but YOLOv8 and YOLOv5-Atn missed less. In the third row, all three models had missed or wrong problems, but YOLOv5-Atn had no misdetection compared with the other two models, which still indicated that YOLOv5-Atn had a better detection effect on residual film.

### 5.3. First-Stage Training Parameters

**Figure 9:**
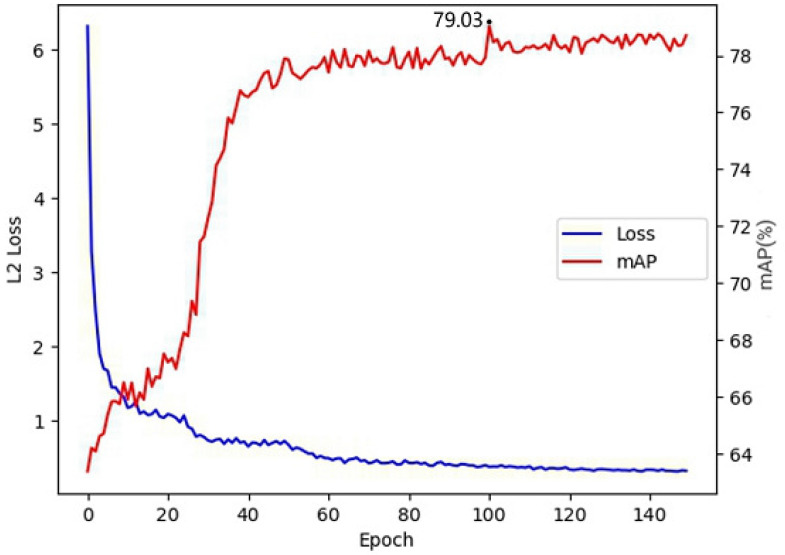
Loss versus map curve diagram.

Experiments were conducted on the epoch sizes chosen for the pre-training process in the first stage, with ten epochs as intervals, counting the loss of 10 to 150 epochs and the mAP corresponding to the prediction results in the second stage, as shown in Figure 9. With the continuous increase of epochs, the loss in the first stage gradually became smaller, and the model converged better, but the mAP corresponding to the second stage was not becoming higher and higher. The mAP of the second stage reached the highest when the first stage epoch reached around 100. This indicated that with continuous training, the model in the first stage had an overfitting problem, resulting in the encoder trained in the first stage being unable to perform at its best when applied to the second stage. It could be concluded from the experiments that the final target detection metrics were highest when the first-stage training was around 100 epochs.

### 5.4. Atrous Convolution Interval Selection

The ASFF module uses atrous convolutions of different sizes to extract features from deep features to obtain feature maps of different scales. Although the ASFF module can adaptively give higher weights to the sizes suitable for residual film, the range of size selection is set manually. In order to better verify the effect of different scales on the ASFF module in feature selection, the convolution kernel size is fixed, and ablation experiments are performed on atrous convolutions with different spacing sizes, as shown in Table 4. From the table, it could be found that the large interval of (4, 6, 8) had the highest accuracy of 77.52% for the atrous convolutional recognition results. However, the recall is the lowest at 67.5%, indicating that many smaller residual film objects had not been recognized. The recognition results of the atrous convolution for the smaller interval of (2, 3, 4) were the opposite of the experimental results for the interval of (4, 6, 8), with the highest recall rate among the experimental results, but the accuracy rate was only 75.47%. It could be seen that although the small scale could better capture the small residual film target, it produced many invalid prediction boxes, resulting in lower accuracy due to the lack of overall context. The experimental results showed that too large or too small intervals could cause different degrees of precision and recall loss. Therefore, another set of experiments with (2, 6, 10) interval sizes was designed, which had both larger and smaller intervals. This made the model balance the recall and precision and keep it at a high level, with a mAP of 78.04%. It was concluded that a suitable atrous convolution interval could help the adaptive weighting algorithm of the ASFF module to better adaptively select based on the feature information of different sizes.

### 5.5. FCA Location Combination

The cross-attention module utilized shallow and deep feature interactions to complete the combination of effective information between space and channel, providing richer feature information for subsequent target detection branches at different scales. In the original network structure, there were three places where shallow features interacted with deeper features, denoted as P1, P2, and P3. We verified the effectiveness of the FCA module by taking one, two, and three interaction parts by permutations and combinations, respectively, and the original concat operator was adopted for the connections not using the FCA module. The experimental results are shown in Table 5. It could be found that the mAP of the model grows along with the increasing number of FCA modules. The highest mAPs were 76.87% for adding only one FCA module, 77.43% for adding two FCA modules, and 78.1% for the experiment in which all the original concat operators were replaced with an FCA module. From the experimental results, it could be concluded that adding each FCA module produces a positive effect, with the help of the interaction of shallow and deep features, effectively eliminating the original interference noise of shallow features, thus proving the effectiveness of the FCA module.

**Figure 10:**
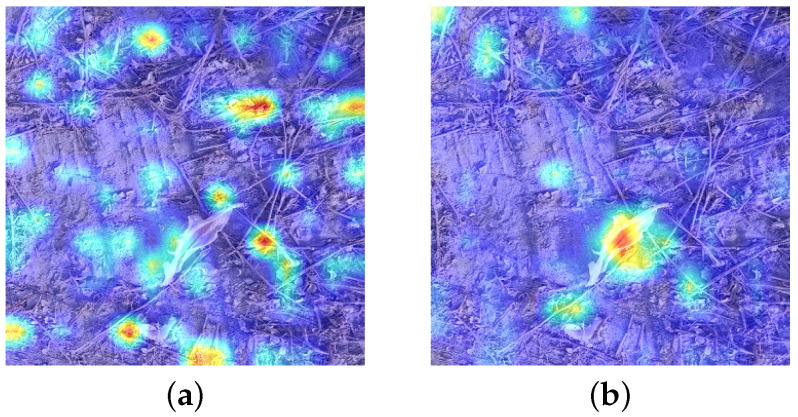
Comparison of feature map visualization. (**a**) Before adding the FCA module. (**b**) After adding the FCA module.

By deep information interacting with shallow information, the FCA module can eliminate the noise information brought by shallow features and better combine image context features. The detection results before and after using the FCA module are visualized using heatmaps, as shown in Figure 10. A darker color indicates that the model pays more attention to the feature information in this region. Before adding the FCA module, the shallow features of the original model paid attention to not only the residual film targets to be detected but also many non-residual-film noises. These noises could have a negative impact when the features were combined. After adding the FCA module, non-residual-film noise attention was significantly reduced. The combined shallow information was filtered through the guidance of deep semantic information, effectively eliminating noise interference. This concentrates the attention on the residual film region that needs to be detected and improves the effectiveness of the contextual information after the features are combined.

## 6. Discussion

There may be challenges in applying the proposed method to larger areas or different agricultural landscapes. Extending the method to larger regions or different agricultural landscapes may require acquiring large amounts of data, which can be more difficult and time-consuming. Significant differences exist in the characteristics of different agricultural landscapes, such as crop type and climate. These differences may be reflected in the appearance of residual film, which represents new challenges for the model.

The model can be combined with the film pickup device of the residual film recycling machine to be applied to the actual collection process of residual film, including the detection of residual film, spatial location information positioning, and other related parts. It can be deployed and integrated into a complete visual recognition system through a development board. After detecting the residual film information in the image, the coordinates of the center point of the prediction box are converted into the spatial position coordinates of residual film, and the coordinate information is transmitted to the film pickup device to realize the recycling of residual film and increase the recycling efficiency of the residual film recycling machine.

## 7. Conclusions

Residual film target detection is significant to agricultural production management and environmental protection. This paper proposed the YOLOv5-Atn model for detecting residual film in agricultural fields, which addressed the problem of diverse morphology and size of residual film. A two-stage training strategy was proposed to optimize the training process for solving the problem of poor model generalization brought by limited data samples. The original SPPF structure was replaced with an adaptive multi-scale fusion module of self-design, which allowed the model to adaptively select the appropriate size of feature maps and improved the recognition accuracy of small targets. A cross-attention mechanism between features was proposed for convolutional networks, which effectively removed interference noise from shallow features utilizing the interaction between shallow and deep features. In order to verify the effectiveness of the proposed model in detecting residual film, experiments were conducted on a residual film dataset acquired in the natural environment. Compared with the original YOLOv5 network, the improved model improved by 3.95%, 2.02%, and 5.39% in mAP, recall, and precision, which indicated that the capability of detecting residual film had been effectively improved. The average detection time per image was 0.0128 s, which could meet the demand for real-time detection in agriculture. The detection effect of the improved model was also better than the latest YOLO series of target detection models, and the detection time was faster, which could provide more reliable support for residual film detection.

In future work, residual film images from different regions and agricultural landscapes will be collected to expand the dataset. In addition, improving detection performance comes at the cost of increased model complexity and parameters. The subsequent research will focus on the model’s lightweight and improving detection speed to facilitate hardware deployment.

## Figures and Tables

**Table 1:** Ablation experiments.

Two-Stage	ASFF	FCA	mAP/%	P/%	R/%	F1/%	mAP@0.5:0.95/%
-	-	-	75.08	72.62	67.94	70.20	44.60
*√*	-	-	76.16	74.78	68.47	71.49	46.41
*√*	*√*	-	78.04	76.67	70.07	73.22	47.74
*√*	-	*√*	78.10	76.70	69.54	72.94	47.98
*√*	*√*	*√*	79.03	78.01	69.96	73.77	48.56

*√* means we use this method, - means we do not use this method.

**Table 2:** Comparison models.

Model	mAP/%	P/%	R/%	F1/%	mAP@0.5:0.95/%
Faster R-CNN	59.50	58.10	44.90	50.65	29.30
YOLOv3	72.60	70.50	52.30	60.05	37.40
YOLOv5	75.08	72.62	67.94	70.20	44.60
YOLOv7	75.60	75.60	68.10	71.65	43.60
YOLOv8	76.40	75.80	70.40	73.00	43.00
YOLOv5-Atn	79.03	78.01	69.96	73.77	48.56

**Table 3:** Model complexity comparison.

Model	FLOPs/G	Model Size/MB	FPS	Inference Time/ms
Faster-RCNN	262.6	315	34.40	29.07
YOLOv3	135.2	234	73.76	13.56
YOLOv5	15.8	13.7	79.52	12.58
YOLOv7	56.3	71.3	48.16	20.76
YOLOv8	28.4	17.5	52.48	19.05
YOLOv5-Atn	36.5	25	77.92	12.83

**Table 4:** Atrous convolution interval selection.

Interval Size	mAP/%	P/%	R/%	F1/%	mAP@0.5:0.95/%
d(2, 3, 4)	77.16	75.47	70.50	72.90	45.85
d(4, 6, 8)	77.19	77.52	67.50	72.16	45.90
d(2, 6, 10)	78.04	76.67	70.07	73.22	47.74

**Table 5:** FCA location selection.

P1	P2	P3	mAP/%	P/%	R/%	F1/%	mAP@0.5:0.95/%
-	-	-	76.16	74.78	68.47	71.49	46.41
*√*	-	-	76.63	74.20	68.65	71.32	45.12
-	*√*	-	76.54	76.73	66.79	71.41	45.95
-	-	*√*	76.87	78.22	66.67	71.99	46.87
*√*	*√*	-	77.15	75.08	68.50	71.64	46.10
*√*	-	*√*	77.43	76.93	68.12	72.26	47.23
-	*√*	*√*	77.11	76.59	68.12	72.11	46.43
*√*	*√*	*√*	78.10	76.70	69.54	72.94	47.98

*√* means we use this method, - means we do not use this method.

## Data Availability

Data available on request from the authors.

## References

[1] Zhao Y., Chen X., Wen H., Zheng X., Niu Q., Kang J. (2017). Research Status and Prospect of Control Technology for Residual Plastic Film Pollution in Farmland. Trans. Chin. Soc. Agric. Mach..

[2] Bu L.D., Liu J.L., Zhu L., Luo S.S., Chen X.P., Li S.Q., Hill R.L., Zhao Y. (2013). The effects of mulching on maize growth, yield and water use in a semi-arid region. Agric. Water Manag..

[3] Bai L., Hai J., Han Q., Jia Z. (2010). Effects of mulching with different kinds of plastic film on growth and water use efficiency of winter wheat in Weibei Highland. Agric. Res. Arid Areas.

[4] Lin T., Tang Q.X., Hao W.P., Wu F.Q., Lei L., Yan C.R., He W.Q., Mei X.R. (2019). Effects of plastic film residue rate on root zone water environment and root distribution of cotton under drip irrigation condition. Trans. Chin. Soc. Agric. Eng..

[5] Yan C.R., Liu S.K., Shu F., Liu Q., Liu S., He W.Q. (2014). Review of agricultural plastic mulching and its residual pollution and prevention measures in China. J. Agric. Resour. Environ..

[6] Pei X.M., Jin X.Q. (2014). Research on farmland residual film recycling mechanization technology popularization and application in Xinjiang. J. Chin. Agric. Mech..

[7] Shi Z., Zhang X., Liu X., Kang M., Yao J., Guo L. (2023). Analysis and Test of the Tillage Layer Roll-Type Residual Film Recovery Mechanism. Appl. Sci..

[8] You J., Zhang B., Wen H., Kang J., Song Y., Chen X. (2017). Design and Test Optimization on Spade and Tine Combined Residual Plastic Film Device. Trans. Chin. Soc. Agric. Mach..

[9] Kang J., Peng Q., Wang S., Song Y., Cao S., He L. (2018). Improved Design and Experiment on Pickup Unit of Spring-tooth Residual Plastic Film Collector. Trans. Chin. Soc. Agric. Mach..

[10] Xie J., Yang Y., Cao S., Zhang Y., Zhou Y., Ma W. (2020). Design and experiments of rake type surface residual film recycling machine with guide chain. Trans. Chin. Soc. Agric. Eng..

[11] Haq M.A., Khan M.Y.A. (2022). Crop water requirements with changing climate in an arid region of Saudi Arabia. Sustainability.

[12] Haq M.A. (2022). Planetscope Nanosatellites Image Classification Using Machine Learning. Comput. Syst. Sci. Eng..

[13] Jiang S., Zhang H., Hua Y. (2016). Research on location of residual plastic film based on computer vision. J. Chin. Agric. Mech..

[14] Zhu X., Li S., Xiao G. (2019). Method on extraction of area and distribution of plastic-mulched farmland based on UAV images. Trans. Chin. Soc. Agric. Eng..

[15] Fu C., Cheng L., Qin S., Tariq A., Liu P., Zou K., Chang L. (2022). Timely plastic-mulched cropland extraction method from complex mixed surfaces in arid regions. Remote Sens..

[16] Hasituya, Chen Z., Wang L., Wu W., Jiang Z., Li H. (2016). Monitoring plastic-mulched farmland by Landsat-8 OLI imagery using spectral and textural features. Remote Sens..

[17] Lu L., Tao Y., Di L. (2018). Object-based plastic-mulched landcover extraction using integrated Sentinel-1 and Sentinel-2 data. Remote Sens..

[18] Sun Y., Han J., Chen Z., Shi M., Fu H., Yang M. (2018). Monitoring Method for UAV Image of Greenhouse and Plastic-mulched Landcover Based on Deep Learning. Trans. Chin. Soc. Agric. Mach..

[19] Yang Q., Liu M., Zhang Z., Yang S., Ning J., Han W. (2019). Mapping plastic mulched farmland for high resolution images of unmanned aerial vehicle using deep semantic segmentation. Remote Sens..

[20] Ning J., Ni J., He J., Li L., Zhao Z., Zhang Z. (2021). Convolutional Attention Based Plastic Mulching Farmland Identification via UAV Multispectral Remote Sensing Image. Trans. Chin. Soc. Agric. Mach..

[21] Zhang X., Huang S., Jin W., Yan J., Shi Z., Zhou X., Zhang C. (2021). Identification Method of Agricultural Film Residue Based on Improved Faster R-CNN. J. Hunan Univ..

[22] Zhou T., Jiang Y., Wang X., Xie J., Wang C., Shi Q., Zhang Y. (2023). Detection of Residual Film on the Field Surface Based on Faster R-CNN Multiscale Feature Fusion. Agriculture.

[23] Wu X., Liang C., Zhang D., Yu L., Zhang F. (2020). Identification Method of Plastic Film Residue Based on UAV Remote Sensing Images. Trans. Chin. Soc. Agric. Mach..

[24] Lin T.Y., Maire M., Belongie S., Hays J., Perona P., Ramanan D., Dollár P., Zitnick C.L. Microsoft coco: Common objects in context. Proceedings of the Computer Vision–ECCV 2014: 13th European Conference.

[25] Ren S., He K., Girshick R., Sun J. (2015). Faster r-cnn: Towards real-time object detection with region proposal networks. Adv. Neural Inf. Process. Syst..

[26] Liu W., Anguelov D., Erhan D., Szegedy C., Reed S., Fu C.Y., Berg A.C. Ssd: Single shot multibox detector. Proceedings of the Computer Vision–ECCV 2016: 14th European Conference.

[27] Lin T.Y., Dollár P., Girshick R., He K., Hariharan B., Belongie S. Feature pyramid networks for object detection. Proceedings of the IEEE Conference on Computer Vision and Pattern Recognition.

[28] Liu S., Qi L., Qin H., Shi J., Jia J. Path aggregation network for instance segmentation. Proceedings of the IEEE Conference on Computer Vision and Pattern Recognition.

[29] Ronneberger O., Fischer P., Brox T. U-net: Convolutional networks for biomedical image segmentation. Proceedings of the Medical Image Computing and Computer-Assisted Intervention–MICCAI 2015: 18th International Conference.

[30] Liu R., Mi L., Chen Z. (2020). AFNet: Adaptive fusion network for remote sensing image semantic segmentation. IEEE Trans. Geosci. Remote Sens..

[31] Vaswani A., Shazeer N., Parmar N., Uszkoreit J., Jones L., Gomez A.N., Kaiser Ł., Polosukhin I. (2017). Attention is all you need. Adv. Neural Inf. Process. Syst..

[32] Liu P., Yin H. (2023). YOLOv7-Peach: An Algorithm for Immature Small Yellow Peaches Detection in Complex Natural Environments. Sensors.

[33] Hu Z., Yang H., Yan H. (2023). Attention-Guided Instance Segmentation for Group-Raised Pigs. Animals.

[34] Chang Y., Li D., Gao Y., Su Y., Jia X. (2023). An Improved YOLO Model for UAV Fuzzy Small Target Image Detection. Appl. Sci..

